# Signalling and the Evolution of Cooperative Foraging in Dynamic Environments

**DOI:** 10.1371/journal.pcbi.1002194

**Published:** 2011-09-22

**Authors:** Colin J. Torney, Andrew Berdahl, Iain D. Couzin

**Affiliations:** Department of Ecology and Evolutionary Biology, Princeton University, Princeton, New Jersey, United States of America; University of Texas at Austin, United States of America

## Abstract

Understanding cooperation in animal social groups remains a significant challenge for evolutionary theory. Observed behaviours that benefit others but incur some cost appear incompatible with classical notions of natural selection; however, these behaviours may be explained by concepts such as inclusive fitness, reciprocity, intra-specific mutualism or manipulation. In this work, we examine a seemingly altruistic behaviour, the active recruitment of conspecifics to a food resource through signalling. Here collective, cooperative behaviour may provide highly nonlinear benefits to individuals, since group functionality has the potential to be far greater than the sum of the component parts, for example by enabling the effective tracking of a dynamic resource. We show that due to this effect, signalling to others is an evolutionarily stable strategy under certain environmental conditions, even when there is a cost associated to this behaviour. While exploitation is possible, in the limiting case of a sparse, ephemeral but locally abundant nutrient source, a given environmental profile will support a fixed number of signalling individuals. Through a quantitative analysis, this effective carrying capacity for cooperation is related to the characteristic length and time scales of the resource field.

## Author Summary

One of the key challenges facing evolutionary theory is understanding how cooperation and communication evolve in social systems. In many situations cooperation leads to higher net benefits to all, but a population of cooperators is vulnerable to invasion from exploitative strategies. When foraging, aiding others through sharing information can lead to an advantage to a collective of communicating individuals. How this behaviour can be maintained and resist invasion without centralized control or policing is currently not clear. In this work, we examine a social foraging system where individuals evolve to signal to conspecifics when they locate a resource. We show that in some environments, cooperative signalling is sustained through a form of indirect reciprocation, as a signalling phenotype is more likely to be the beneficiary of a signal from a conspecific in the future. This effect naturally occurs as a result of the foraging dynamic and, depending on the environment, such as how resources are distributed and how difficult they are to track, will compensate for relatively large costs of signalling. Through simulations and a simplified model we examine the parameters driving this process and identify the mechanisms required for cooperation to evolve in such a system.

## Introduction

In many systems subject to evolutionary pressure, there exists a discrepancy between behaviour that is adaptive at the individual level and that which would be most beneficial for higher levels of social or biological organization. When individual self-interest runs counter to the best interests of the collective, it can lead to what is known as the *tragedy of the commons* [1]. While a cooperative, enlightened approach results in higher average net benefits to all, an individual that contributes nothing but benefits from the behaviour of others will hold an advantage. This fitness differential allows the invasion of non-cooperators, to the detriment of the collective [2].

Despite this issue, examples of altruism and cooperation abound in the natural world [3]. Indeed cooperative behaviour and the suppression of competition for the benefit of higher level entities are hallmarks of the major transitions in evolution [4]. Several explanations have been proposed for this apparent paradox [5], the most pervasive and all-encompassing being Hamilton's notion of inclusive fitness [6]. However, open questions remain, notably concerning the relative importance of different drivers of cooperation amongst non-kin [7], the effects of synergistic interactions on the evolutionary dynamic [8], [9], and how to engender optimal, cooperative solutions in artificial or social systems [10], [11].

Locating and exploiting resources is an ever present challenge for all organisms, and it is an area where cooperative strategies can greatly improve the probability of success. Social foraging theory has shown animals in groups are able to acquire more information about their environments than if they were to forage alone [12]–[15]. Search efficiency and the processing of environmental cues may consequently be improved [16]–[19], while sharing the located resources with conspecifics dissipates the risk associated with unsuccessful foraging attempts when conditions are unpredictable [20].

Effective and honest communication in these situations would clearly improve foraging efficiency since it provides individuals with an additional level of reliable information [21]. However, while it is clear to see how individuals would evolve to take advantage of the inadvertent social information provided by others [22], understanding the evolution of honest communication represents a further challenge [23], [24].

The study of information in an ecological context is an active and important area of research, encompassing learning, communication, exploitation through informational parasitism, and strategic social interaction [25], [26]. The seminal idea for this field is the information centre hypothesis (ICH) proposed by Ward and Zahavi [27]. They suggested that communal roosts, breeding colonies and other bird assemblages have evolved primarily for the purpose of sharing information. While this work has inspired many further investigations, it has generated some criticism [28], notably due to its reliance on a group selectionist argument to explain costly flight displays [29].

As an alternative to the ICH, Richner and Heeb proposed the recruitment centre hypothesis (RCH) [30], which argued that since foraging in groups often provides some benefit (e.g. increased predator vigilance, access to defended resources), aggregations of conspecifics provide successful foragers with a recruitment centre from which to recruit others in order to exploit located resources. However this hypothesis also relies on an implicit group selection argument [31], [32], since it does not explain how the collective resists degradation due to informational parasitism.

Several studies based on evolutionary game theory and numerical simulations [32]–[35] have shown both hypotheses are potentially correct, depending on the ecological circumstances, such as the benefits of group foraging [33], [36], the finder's share, i.e. the advantage of locating the resource first [32] and the temporal dynamics of the resource. These studies also emphasize the distinction between inadvertent social information and the active recruitment of conspecifics, and they suggest the ICH is an appropriate explanation for the evolution of social aggregation when information is shared inadvertently [37], whereas when costly, active communication is involved, there must be an offsetting advantage, as is the case for the RCH.

Active recruitment of conspecifics to resources is observed in several species, and when not attributed to indirect fitness benefits (see e.g. [38]), is often associated with a manipulation of the environment. For example, by increasing the local density of foragers an individual may in fact reduce its own risk of predation [39], [40], or be able to gain access to defended resources [41], [42]. In some ephemeral environments it has been noted that food calling may be beneficial since it enables the tracking of a resource. This behaviour has been observed in cliff swallows [43], [44] where it occurs when the food source (in this case an insect swarm) is advected by moderate winds. In this situation acting cooperatively could result in a higher level functionality as signalling enables a collective-level response to the environment through the effective tracking of the insect cloud. However, non-signallers are able to exploit other signallers without incurring the associated costs arising, for example, through the energetic costs of producing the signal.

The purpose of this work is to investigate the conditions where signalling can be maintained due to the nature of the resource environment. Through a numerical study of evolution in a two dimensional turbulent environment we show that, within a certain region of parameter space, a cooperative signalling strategy is stable. A reduced model that retains the essential features of the full simulation is then analyzed and the mechanisms that drive the evolutionary dynamic are explored.

## Model

The underlying biological and physical processes that shape environmental conditions often result in patchy and heterogenous landscapes [45], [46], where resource distribution is highly variable. Additionally, stochastic advective forces by their nature, lead to a stretching and folding of the resource, resulting in localized high concentration regions with filamental structures [47].

The summary effect of these processes is the presence of steep local gradients in nutrient concentration. Fluctuations and stochasticity in an organism's position relative to the resource, are therefore capable of significantly affecting both nutrient uptake and perception of the resource. Perturbations, either due to the nature of the advective carrier flow, or to the random motion of the organism, may result in sharp decreases in the experienced resource concentration and the loss of an unexploited food patch. In this situation social interactions can be greatly beneficial as the effective sampling size of the organism is increased, and when a resource is lost it may be relocated by following others [13], [14], [16]. Conspecifics in this case may be considered as a source of information [25], [48], [49] and the behaviour of other individuals can be modified to either enhance or impair this information [50], [51].

In our model individuals forage within a chaotic environment and freely evolve the ability to do so cooperatively, by signalling to others when they have located a region of high nutrient availability. It is assumed this is an active behaviour as opposed to inadvertent social information (ISI) [22], and also that it elicits an appropriate response from near neighbours that are seeking the resource. In this scenario ISI as described in [22], may be a precursor to the active recruitment of others, since as shown below, a conspicuous response to locating a resource may well be adaptive.

### Foraging environment

To generate a realistic stochastic environment a synthetic turbulence model was used [52]. This approach randomly evolves the phase and amplitude of the Fourier modes of the carrier flow, and through a wavelength dependent noise intensity is able to create an isotropic turbulent flow with a prescribed energy spectrum. Here we use the Kárman-Obukhov spectrum [53], however the results presented are independent of the statistical properties of the carrier flow as demonstrated in section.

The flow field is used to advect a concentration field 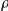 (representing a nutrient source), that is advected, added into the system at a constant rate and subject to exponential decay. Hence
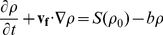
(1)where 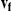 is the flow velocity, and 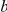 is the decay rate. 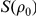 is an advected nutrient source defined as
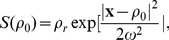
(2)so it is added at a rate defined by 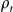, over a width defined by 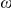 and its centre is advected according to 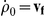. By introducing the nondimensional variables 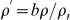 and 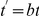, Eqn. 1 becomes (after dropping primes),
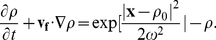
(3)


**Figure 1:**
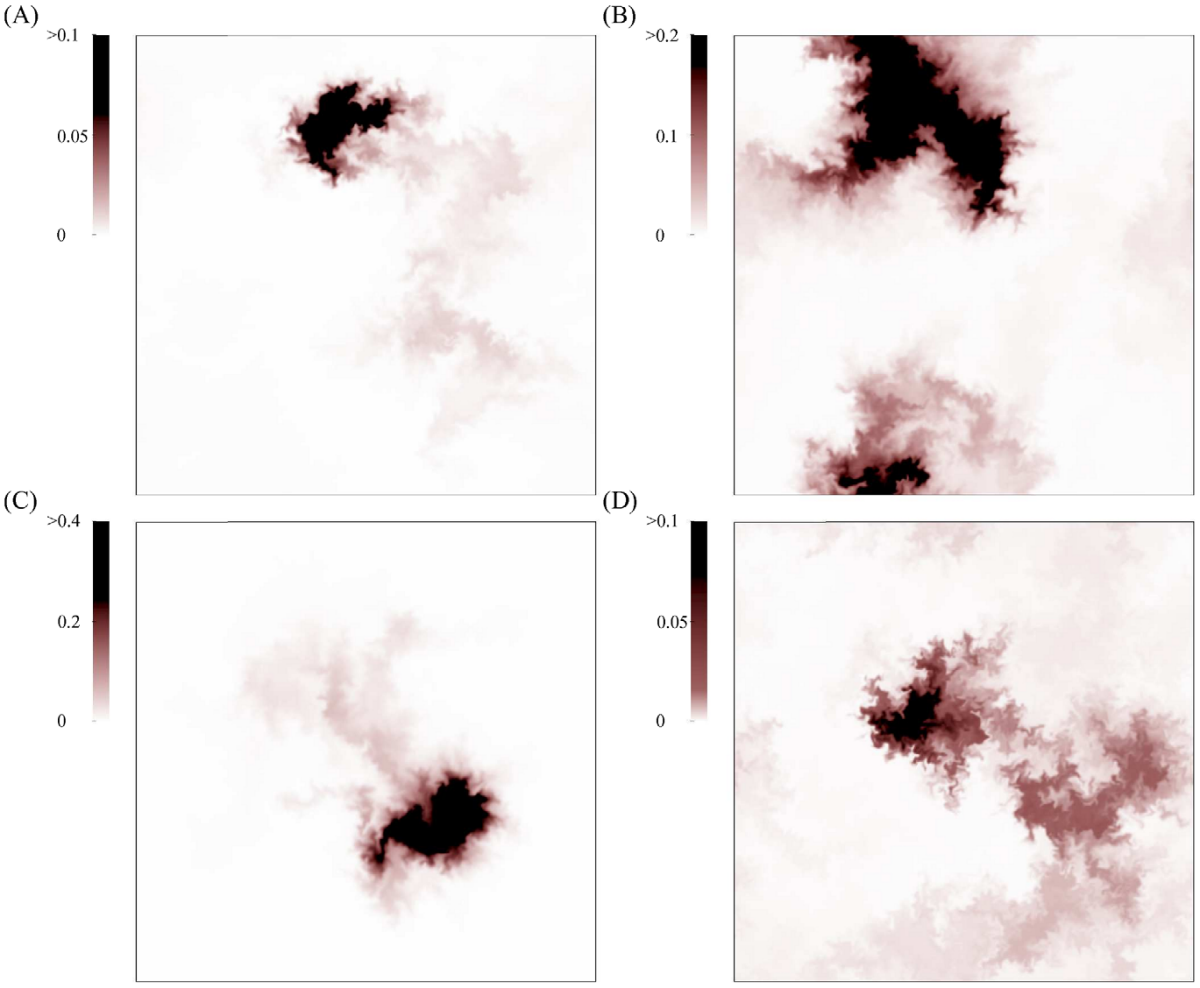
Snapshots of resource field for different parameter values. (A) Size of source, 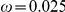, mean absolute velocity 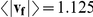, (B) 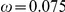, 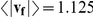, (C) 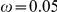, 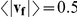, (D) 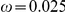, 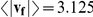. Colour bars show resource concentrations.

We fix the length scale of the largest energy mode to be equal to the system size 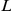 and rescale so that the environment is simulated on the unit torus. This leaves two parameters which determine the characteristics of the resource field, the average flow velocity, 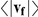 and the width of the exponential source term 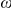. In Figure 1 snapshots of the environment for various parameter values are shown.

### Behavioural rules

Individuals foraging in the generated environment follow simple behavioural rules corresponding to two distinct and discrete strategies. The two categories of individual are signallers (S), a strategy that may be equated to the cooperate strategy of traditional game theoretic models, and non-signallers (NS), which equivalently are considered analogousto defectors. When signallers locate a favourable nutrient region, they actively recruit others through some form of communication. If an individual is within range of a signal and is not currently located in a preferred region, this individual becomes attracted to the source of the signal.

At the individual level no search strategy exists and no form of taxis occurs. Instead a solitary individual performs a correlated random walk through the environment at constant speed, so that their average nutrient uptake is equal to the mean concentration. While there are many asocial strategies that result in a nutrient exposure greater than this mean value (see e.g. [54]–[56]), we select this as the baseline asocial performance. Since we are interested in the relative improvement provided by cooperation, the baseline asocial uptake rate is arbitrary and incorporating a more intelligent asocial response would be equivalent to a rescaling of the cost function. As supplementary material (Text S1 and Figure S1) we include an investigation of the effect of an asocial search strategy, and show that the qualitative features of the results described below are unaffected.

In our model all individuals are advected by the flow and propel themselves at a constant speed along their axis of orientation,
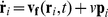
(4)where 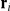 is the spatial position of individual 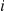, 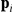 is its orientation, and 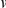 is the constant self-propulsion speed. Two further variables define an individual's state, a normalized current concentration value,
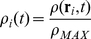
(5)and a state of signalling or not 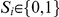, where 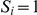 indicates individual 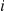 is giving a signal that it is located in a high nutrient region, which is then perceived by all neighbours within a certain interaction range.

The signal and response dynamic is stochastic with the probability of performing a certain behaviour determined by both phenotype and external conditions. At each time step, the probability of emitting a signal is
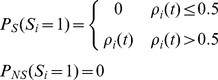
(6)for the respective cases of a signaller and a non-signaller. The probability of responding to a signal is the same for both phenotypes and depends only on the experienced nutrient concentration (i.e. an individual experiencing a high resource concentration will more readily ignore a signal),
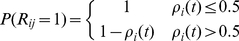
(7)where 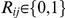 defines whether individual 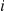 will respond to a signal from 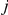. Based on this interaction a preferred direction of travel, 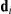 is defined,
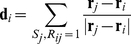
(8)so that an individual turns toward the average location of the individuals that are signalling and that it has decided to respond to.

It is worth noting at this point, individuals only interact when a signal has been given. In the absence of signalling all foragers are effectively invisible. While responding to non-signalling conspecifics may be beneficial [25], active recruitment will provide an additional benefit for the collective, hence by assuming only signalling individuals are attracting, we focus solely on this increased benefit. Since in our model the nutrient resource is not consumed and individuals are represented by point particles, this effective invisibility extends to preclude issues of crowding and consumption. These effects are then avoided, and the cost experienced by signallers is incorporated into a single parameter that discounts their uptake rate.

The signalling cost may arise as a result of various factors, the most obvious being the energy expended in producing the signal. While this may seem slight, the energy budget for free-living birds is often finely balanced [57]. Signalling also has the potential to attract the attention of predators, however this may be countered by an increased dilution and/or confusion effect [58], [59], and the relative significance of these effects is highly context dependent. It is assumed that competition through consumption is not an issue, i.e. the product of meal size with number of foragers is less than the total resource available, and individuals may modulate their signal range in order to ensure this is the case. Despite this, since access to the resource is time limited, there may well be a cost from interference effects or crowding.

Regardless of the nature or source of the immediate cost of signalling, in a well mixed, highly mobile population, any behaviour that benefits others is essentially costly since it will increase local competition for mates, territory or preferred breeding sites. The act of signalling must therefore be understood in the context of the direct advantage it conveys to the actor.

## Results

### Evolutionary simulations in a complex environment

The within generation process is defined by the environment and the behavioural rules outlined above. The foraging success of each individual is dependent on their phenotype, the behaviour of others and the statistical properties of the resource field. Simulations of the foraging process were performed, then a roulette wheel algorithm was used to select individuals to contribute to the next generation according to their fitness. Here fitness is defined as normalized foraging success less the cost paid through signalling. Cost is levied at a constant rate, not on a per signal basis, although both are statistically equivalent.

**Figure 2:**
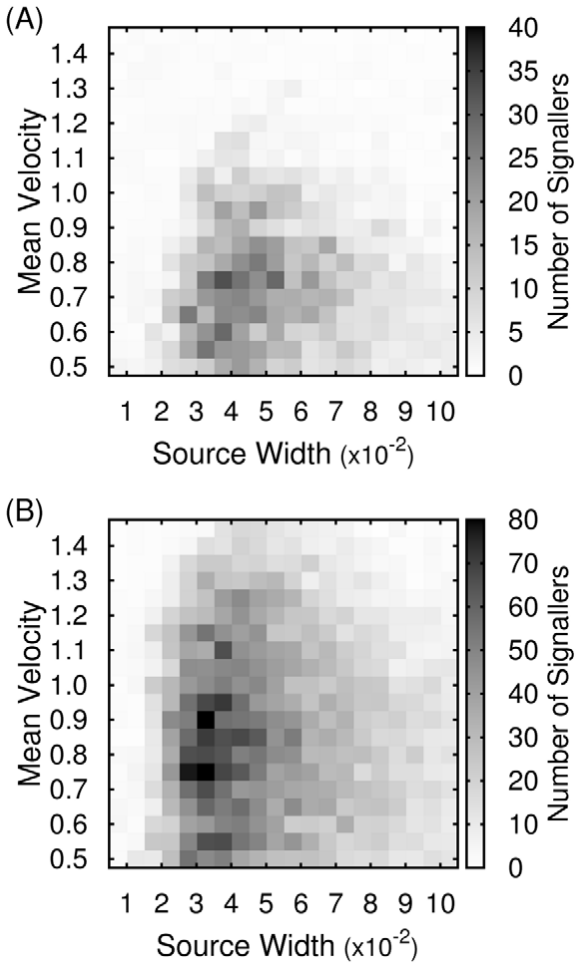
Evolutionary equilibrium number of signallers. Results are for a range of mean velocities, 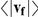 and source widths 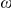. Cost values are for, (A) 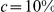 and (B) 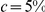.

**Figure 3:**
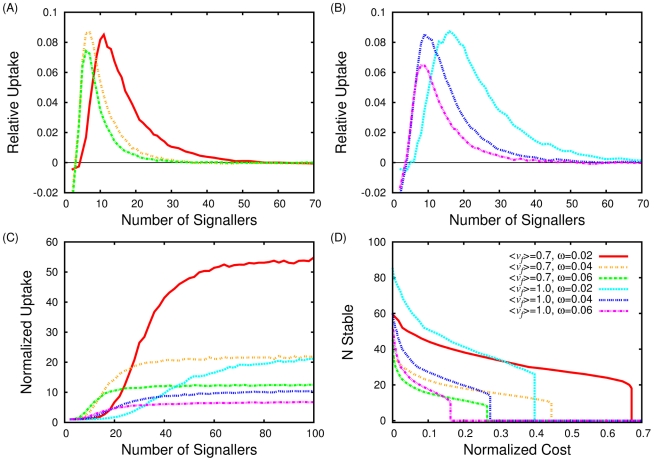
Resource uptake rates for the turbulent simulations. (A–B) Relative uptake, 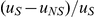, between signallers and non-signallers as a function of signaller number for various values of source width 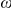 and (A) 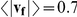, (B) 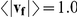. (C) Average uptake, 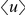, normalized by mean resource concentration, 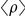, for various values of 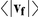 and 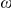. (For the asocial strategy 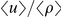 is unity). (D) The nontrivial (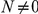) evolutionarily stable number of signallers as a function of the cost of signalling for various values of 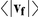 and 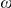 (cost is shown as a percentage of the mean resource concentration 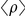). Legend for all figures as (D).

The steady state absolute number of signalling individuals for a range of parameter values are shown in Figure 2, for two different cost values. (The cost is defined as a percentage of the mean resource value to ensure consistency across varying patch sizes.) For these simulations an initial seeding of 8 signalling individuals was used in a total population of 512. Although reasonably arbitrary, this initial number is required as a small critical threshold of signallers must be present for the strategy to be viable (as illustrated in Figure 3).

These plots demonstrate the existence of a fixed density of signallers that is robust to invasion by the non-signalling phenotype and that is dependent upon the statistical properties of the resource field. The region of parameter space which supports the cooperative phenotype is characterized by intermediate patch size and flow velocity. In this regime the resource field is not widely distributed or well mixed, and there exists a high variance in concentration. When the patch source size, 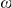 is very small, uptake is low for all strategies and the additional cost drives signallers to extinction.

In Figures 3(a) and 3(b) we plot the relative uptake of signallers (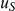) over non-signallers (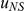) for different signaller densities. Since we are encapsulating the costs of signalling within a single parameter, effects such as the increased competition for the resource are not explicitly included, thus the presence of the non-signalling phenotype has no effect on others and their density is not relevant. Since the number of non-signallers is therefore arbitrary, it is the absolute number of signallers in the population that is the quantity of interest, rather than their percentage. It should be noted that while the relative frequency of cooperators and non-cooperators will affect the speed of evolution, the direction of selection and the location of points of evolutionary stability are determined only by the absolute number. The increase in uptake experienced by a group of signallers as their number increases is shown in Figure 3(c).

From these data it can be observed that once a threshold number of signallers is reached, the strategy outperforms non-signallers. Under selection pressure this advantage leads to an increase in the number of individuals adopting the signalling strategy until a certain equilibrium density is attained. At this point both discrete strategies have on average equal fitness and an evolutionarily stable polymorphic population exists. The value of this stable density is a function of the cost attributed to emitting a signal, and the temporal and spatial correlation lengths of the resource. From Figure 3(d) it can be seen that relatively large increases in cost have only marginal impact on the evolutionarily stable density, due to the steep gradients in differential uptake that occur as this density changes.

The reason for this outcome lies in the manipulative effects of the signalling individuals. Effectively a signaller increases the local density of conspecifics in its immediate vicinity, meaning it is more likely to subsequently benefit from the behaviour of other signallers in the population. To understand how the properties of the resource field influence this dynamic, we now introduce a simple model representation that is amenable to an analytical treatment and a fuller exposition of the underlying mechanisms.

### Numerical and analytical study of a reduced model

For analytical tractability we now consider a reduced, but qualitatively equivalent model, in which individuals follow analogous behavioural rules, but instead forage in an environment with a simple resource distribution and no advective forces. In this environment (see Video S1 for an animation of the model), the resource is represented by a single circular patch of radius 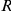 that encloses a region of uniform concentration. This patch persists for time 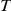, before periodically moving to a new, randomly selected location.

Individuals move at constant speed and are unable to stop when locating a resource patch. This constraint is enforced so that the model is consistent with the full simulations described above. When chaotic, advective forces are present an individual cannot simply maintain its position in a region of high resource concentration; instead maintaining this position is an active process that requires constant processing of environmental and social cues or signals. Since in our reduced model there is no advection, we capture this effect by imposing the constant velocity condition, effectively ensuring there is a non-zero relative velocity as is the case when the resource and/or individuals are subject to stochastic advective forces.

In combination, the constant individual velocity and intermittent relocation of the resource patch capture the dynamics of the full model. The spatial variance of the resource determines the range over which uptake rates can vary, i.e. more localized, high concentration regions lead to a greater difference between an effective search strategy and random motion. However, the role of the temporal dynamics is more complex. The aim of this section is to understand this role by isolating the essential features of the full model. We do this by coarse-graining the spectrum of the turbulent velocity fluctuations into two processes that operate at different time scales. The first is characterized by the frequent occasions on which the resource is lost by the collective. This is equivalent to the resource relocation in the reduced model, and to large scale fluctuations in the full model. The second process operates over the short term and involves the loss of the resource by individuals, imposed by forcing individuals to move through the patch in the reduced case, and by the constant, small scale fluctuations in the full simulations.

**Figure 4:**
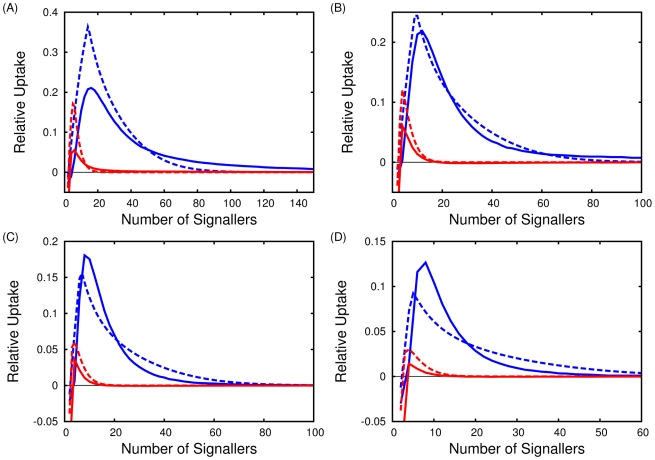
Relative uptake between signallers and non-signallers as a function of signaller number. Each figure shows a unique patch persistence time, (A) 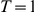, (B) 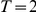, (C) 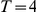, (D) 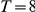. Blue lines are for patch size 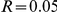, red for 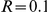. Solid lines show numerical results, dashed lines show the equivalent analytic solution.

Our model therefore has only two relevant parameters, the spatial correlation length of the resource, 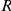 and its temporal correlation, 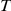. Numerical simulations were performed for various combinations of 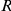 and 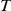 and the relative uptake of signallers compared to non-signallers is plotted in Figure 4. The results qualitatively match the full simulations and show that the requirements for the evolution of signalling are that the resource is localized, requires cooperation in order to be tracked effectively, and is intermittently lost.

To more quantitatively understand this process four steps are required, each involving a certain degree of approximation, but which in combination provide a heuristic and intuitive explanation of the underlying mechanisms which link the statistical properties of the environment to the evolutionary dynamic. In summary these steps are

1. Firstly time is discretized and it is assumed the future state of the system depends only its state in the current time interval and not on previous history.
2. Secondly, transition probabilities are determined for an individual to enter or exit the resource patch depending on whether a signal has been given or not.
3. Next the dynamic is divided into two distinct temporal regimes. The first occurring immediately after the resource has been lost and continuing until a stable cohort of signallers have located it. The second regime begins when this stable cohort emerges and continues until the resource again relocates.
4. Finally, the relative advantage of the signalling strategy is calculated during the transient first regime. During the second regime both strategies perform equally, hence the overall advantage to signallers is calculated by weighting the transient advantage according to the relative lengths of the two regimes.

#### Temporal discretization

In the analysis that follows we assume events occur at discrete time intervals. Within a given interval an individual forager may make the transition from a state of being external to the food patch to being within the resource, and vice versa. The most natural choice for the length of this time step is the time required for an individual to cross the resource patch at its widest point, 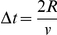 where 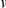 is the speed of an individual. The probability an individual enters the patch within this interval can then be calculated for both the case when no signaller is present, that is the individual enters by chance, and when a signal has been received.

Further to this, it is assumed the probability of the system transitioning to a given state at the next discrete time interval is dependent only on the current state, and not on any previous history, i.e. the process exhibits the Markov property [60].

#### Transition probabilities

We define 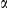 as the probability an individual enters the resource patch in the absence of any signal and 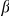 as the probability of entering given a signaller is present within the patch and indicating its location. By definition 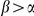.

Since the time interval, 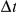 is defined as the minimum time taken to travel a distance of 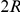, an individual may only enter the food patch at the next time step if it is within this distance of the circumference of the patch. If a signal is given, any individual in the outer ring of width 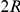 that surrounds the patch, will move directly towards the source of the signal and enter the patch at the next time step.

Therefore, the probability to enter the patch given that a signaller is present, 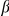, is equal to the probability of being in the outer ring. Since our environment is of unit area this probability is equal to the area of the annulus defined by the two concentric circles,
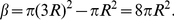
(9)


However, if no signaller is present in the patch, an individual within the outer ring may still enter the patch within the next time step due to its random motion. This probability, 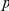, is a function of the distance to the edge of the resource, 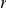, which ranges from 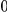 to 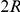. We assume that an individual infinitely close to, but not within the patch, will enter and pass through with probability 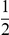, i.e. it will either more into or away from the patch. While in the limiting case of being at the edge of the outer ring (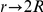) this probability goes to 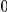. Further, we assume that 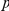 varies linearly between these two limits as a function of the distance to the resource, leading to
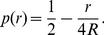
(10)


To find 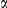, the probability to enter the patch given no signaller is present, we calculate 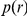 multiplied by the probability to be at 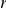, and find the average of this value by integrating over the area of the outer ring. This leads to
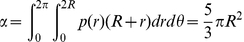
(11)


#### Division into two regimes

We note two distinct regimes in the dynamics of the system. When the patch first appears in a new location it typically has a high probability to be unoccupied or only intermittently located before being lost again. Eventually a small cohort of signallers will form upon the patch, where they are able to leverage their mutual interactions to stay on the patch and remain there until it moves. In this second phase signallers and non-signallers alike arrive at the same rate, have little chance of losing the patch and enjoy equal resource uptake.

**Figure 5:**
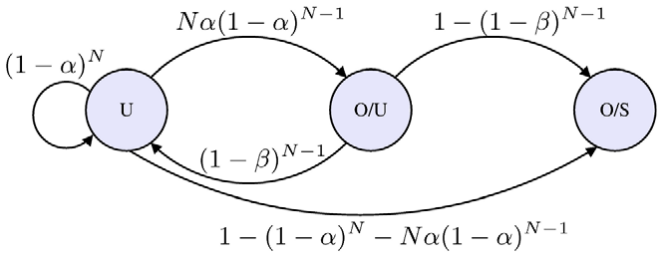
The three state approximation shown with transition probabilities. State 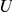 (unoccupied) in the diagram corresponds to state 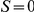 in the equations. Similarly state 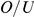 (occupied, unstable) is 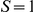 and 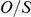 (occupied, stable) is the absorbing state, 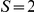.

However, the nutrient uptake during the first regime is greater for signallers as compared to non-signallers. For this reason we calculate the relative length of these regimes. To do so, we consider a three state system, 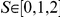, shown in Figure 5.

The transient phase is divided into two states, the first 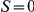 corresponds to the patch being unoccupied (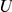) by any signaller, while in the next state, 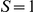, the patch is occupied but unstable (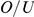), that is a single signaller is present but it will move away from the patch unless another signaller enters.

The final, absorbing state, 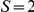, corresponds to the second regime, when two or more signallers are continually in the patch (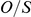). The transition probabilities from state 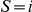 to state 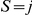, 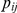, are calculated and shown in Figure 5. Note 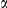 and 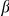 are, respectively, the probability to enter the patch by chance, or to enter when a signaller is present as calculated in the previous section.

State 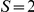 is an absorbing state, and the mean first passage time to arrive in that state, 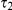, will give us the characteristic time the system typically spends in the first regime,
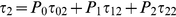
(12)where 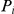 is the probability to be in state 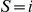 when the patch first appears in a new random location and 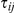 is the mean first passage time from state 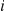 to state 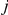.

We calculate 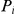 by assuming a random distribution of individuals on the reappearance of the nutrient patch, while the mean first passage times are easily found from the transition probabilities shown in Figure 4 and a recursive formulae obtained from the theory of Markov processes [60].

#### First regime occupation probabilities

Now that we have solved for the length of the transient phase we turn to the relative uptake between signaller and non-signallers during this regime. We reduce this regime to a two state system with the patch initially unoccupied, and focus on two representative individuals employing each strategy.

It is assumed that once inside the patch a single individual will leave at the next time step unless another signaller enters. Therefore a signaller will leave if not able to attract at least one of the other 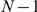 signallers, ie. with probability, 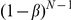. One or more signallers will be attracted to the patch with probability 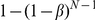, in which case the focal signaller will remain there. The transition matrix for the focal signaller entering and leaving the patch is,
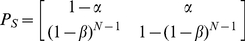
(13)where element 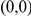 is the probability that given the individual is away from the patch it will remain in this state, element 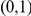 is the probability of locating the nutrient patch at the next time step, 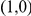 the probability to lose the patch given it has been located, etc.

We can construct analogous matrices for the non-signaller, but the probabilities are dependent on whether or not a signaller is already within the patch,
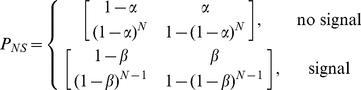
(14)From Eqns. 13 and 14, the distinction between the strategies can be seen. Both require a signal from another to remain within the patch, but by not giving the recruitment call the non-signaller is relying upon a signaller entering by chance, hence the difference between 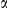 and 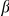 is the key driver of the relative advantage of the signalling strategy.

Using these transition matrices we solve for the equilibrium patch occupation probabilities, 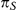 and 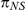, for signallers and non-signallers respectively, which gives for signallers,
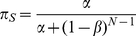
(15)and, for non-signallers,
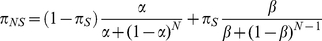
(16)


The occupation probability for the patch is proportional to the mean uptake of resources for the individuals during the transient phase. We now weight the occupation probabilities by the relative time spent in the transient regime, 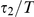, to arrive at the overall uptake differential between the two strategies,
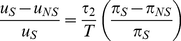
(17)


Figure 4 shows Eqn. 17 plotted as a function of 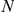, alongside the numeric results of the reduced model. Our analytic approximation matches the data for a range of temporal and spatial correlation lengths, captures the qualitative trend of the full model, and illustrates the role of the environmental parameters on the evolutionary simulations.

## Discussion

Many organisms share information, either inadvertently or through some form of active communication. When communication is honest and appears to benefit the recipient of the information but not the donor, it is often considered a form of cooperative behaviour. The purpose of this work is to further our understanding of the mechanisms that lead to the evolution and stable existence of such behaviour.

 One example of recruitment signals occurring in nature is the food calling observed in species of the cliff dwelling swallow, *Hirundo pyrrhonota*, when feeding on advected clouds of insects. Recruitment calls are given only in certain environmental conditions when the wind speed is at an intermediate level and it has been postulated that calls are given to improve the foraging success of the recruiter by enhancing its ability to track the resource [43], [44].

We have shown that signalling that a food source has been located is indeed an adaptive strategy. We go beyond speculation and provide a mechanistic explanation for the evolution of this behaviour. Signallers raise the local density of fellow signallers around them, this enables a collective response to the resource and hence strongly influences foraging performance. When conditions are appropriate, this effect is sufficient to offset relatively large costs imposed on signalling.

The simulations of the full turbulence model we present demonstrate the existence of a region of parameter space in which the cooperative, signalling strategy is stable. At first glance the key mechanisms that create this outcome are unclear, but by introducing a reduced model we relate the properties of the environment to the stable density of signallers. This reduced model effectively displays two separate time scales. By enforcing each individual to move at a fixed speed, a time scale at which the resource is lost is created, thereby giving an advantage to cooperation. This time scale is defined by the average time taken to traverse the resource, if a signal from a conspecific isn't received within this time frame, the resource will be lost.

The second time scale is defined by the time between relocation events, when the resource is entirely lost. In the full simulations, this is equivalent to the infrequent events when large velocity fluctuations cause all individuals to lose track of the resource. The reduced model effectively has a bimodal distribution of stochasticity, while in real dynamical systems a continuous spectrum exists, but this difference is not important. What matters is that on a shorter timescale, cooperation is beneficial (how beneficial depends on the local variance of the resource i.e. its spatial correlation), whereas infrequent, but more severe fluctuations, put an effective time limit on the period signallers may be exploited, weighting the benefit towards those that contribute to the collective effort early, and thus restricting the evolution of a defector strategy.

These mechanisms can be related to the processes involved in other studies of information use in ecology, notably those concerning the information or recruitment centre hypotheses for colonial living birds [27], [30]. While these studies consider a form of central place foraging the requirements for active communication to evolve are analogous. In all of these studies, as in our model, there is a cost associated with recruitment, for example through the energetic costs of returning to the nest or of performing pre-departure displays. For information transfer to evolve as an active, adaptive behaviour there has to be a group level benefit, while to be robust to invasion from non-cooperating strategies there must be a finder's advantage for the communicating strategy [32].

To facilitate comparison to such works (e.g. [25], [32]) we note some key similarities and differences to our model. Common properties are the presence of a cost associated to recruitment, an advantage to foraging in a group, the presence of a sparse, abundant and ephemeral resource distribution, and that the relative advantage to discovering the resource first is dependent on the timescale over which the resource lasts.

Important distinctions are that the finder's share is conditional on recruitment, hence recruitment does not only reduce the information producer population as in [32] but instead enables the transient increased uptake. Secondly, the group advantage is not acquired when other individuals regardless of phenotype are present, such as is the case when it is provided by access to defended resources [33], [36] or via local enhancement [61], but is instead dependent on the presence of individuals with the cooperative phenotype.

Our results suggest signalling strategies may have evolved in a wide range of scenarios. Diffuse resource fields scattered by advective flows, as in our full turbulence model, are ubiquitous throughout aquatic and aerial environments. Scavengers and decomposers may face a similar challenge when locating and staying with resources that may be lost due to movement by flows or larger organisms, or through displacement by dominant competitors if insufficient conspecifics are present. Further, organisms constrained to provide information to conspecifics through cues, such as strongly electric fish which use electric fields to capture their food, or other organisms inadvertently displaying stereotyped feeding behaviour (including, for example, hunger or dominance displays [42]), may be predisposed to signalling and therefore an evolutionary stepping stone to active recruitment and communicating, cooperative social groups.

## Supporting Information

Figure S1
**Relative uptake between signallers and non-signallers as a function of signaller number for various values of the asocial search parameter.** Source width, 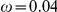, 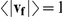. Inset: Increase in uptake for lone individuals as a function of search parameter, 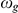. Uptake value is normalized by the mean resource concentration.(TIF)

Text S1 **Individual search behaviour.** Further analysis of the effects of including an individual search strategy in the evolutionary model.
(PDF)

Video S1
**Animation of the foraging dynamics for 48 signallers and 48 non-signallers.** Reduced model parameters 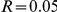, 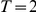. Uptake is approximately equal between the two strategies; signaller density is high and the resource is continually located and exploited.(AVI)
