# Supplementary figures and images for: Signalling and the Evolution of Cooperative Foraging in Dynamic Environments
Source: PLoS Comput Biol. 2011 Sep 22;7(9):e1002194. doi: 10.1371/journal.pcbi.1002194 (PMC3178622; doi:10.1371/journal.pcbi.1002194)

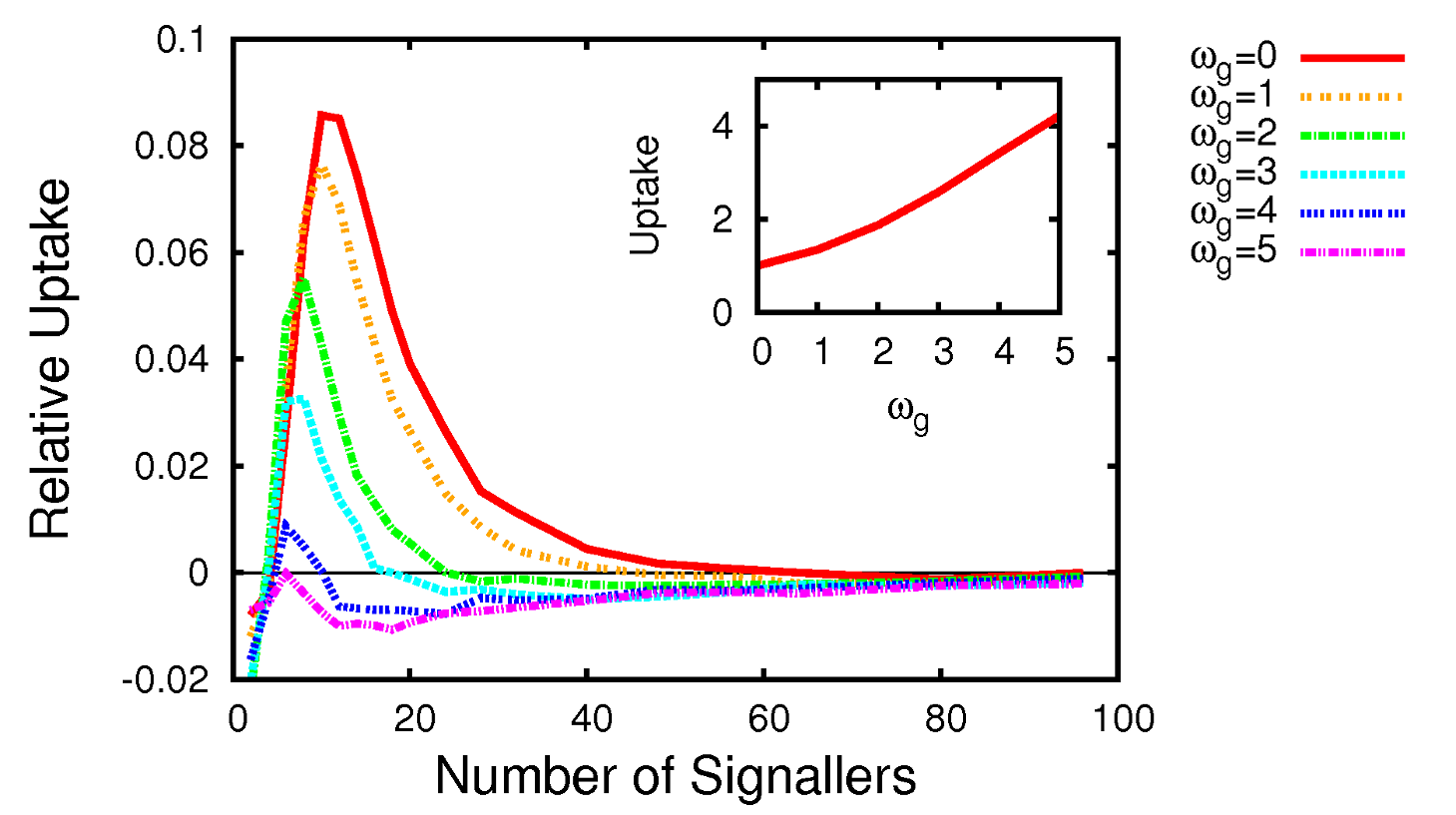

Supplement: Figure S1 — Relative uptake between signallers and non-signallers as a function of signaller number for various values of the asocial search parameter. Source width, , . Inset: Increase in uptake for lone individuals as a function of search parameter, . Uptake value is normalized by the mean resource concentration. (TIF) [file pcbi.1002194.s001.tif]
