# Supplementary material for: Signalling and the Evolution of Cooperative Foraging in Dynamic Environments
Source: PLoS Comput Biol. 2011 Sep 22;7(9):e1002194. doi: 10.1371/journal.pcbi.1002194 (PMC3178622; doi:10.1371/journal.pcbi.1002194)
Supplement: Text S1 — Individual search behaviour. Further analysis of the effects of including an individual search strategy in the evolutionary model. (PDF) [file pcbi.1002194.s002.pdf]

Supplementary Text S1

Individual level search behaviour

In order to clearly emphasize the role of social interactions on foraging success, the results presented in the main manuscript assume no individual level search strategy exists. This is justified since individual behaviours that increase nutrient uptake can be incorporated through a rescaling of the cost function, so long as there remains an advantage to be gained by collectively employing the cooperative strategy. By introducing an individual foraging behaviour the uptake of both phenotypes increases and the importance of signalling is reduced. If there is a commensurate reduction in the cost of signalling then we will recover the evolutionary dynamics observed in the absence of an individual search mechanism.

To demonstrate this effect we introduce a gradient detection capacity into the foraging simulations within the full turbulent environment. This is achieved by assuming each individual is able to detect the instantaneous local spatial gradient in the resource field ρ. The individual then turns toward this direction with an angular velocity defined as

$$\omega_g \left(1 - \exp\left[-|\nabla\rho|^2\right]\right). \tag{1}$$

Eqn. 1 defines a saturating function of resource gradient magnitude, with a maximum value determined by the gradient response parameter ω_g. Shallow gradients in the concentration illicit a very weak response, whereas large gradients are more readily determined and individuals turn rapidly towards the direction of steepest ascent. In the inset of Figure S1, the increased uptake as a function of ω_g is shown for lone individuals. Hence ω_g is the parameter that controls the effectiveness of the asocial strategy, and the degree to which individuals are able to track the resource in the absence of signalling. It should be noted that while a mechanism of this form is effective, it is by no means a simple task to accurately measure the gradient of a dynamic resource field. In many real systems (both artificial and natural), a measurement of this kind would not be possible, and this is especially true when the resource is not continuously distributed, i.e. in the case of aggregated but discrete food particles.

In Figure S1 the effect of introducing an individual level search behaviour is shown. These results show that as the ability to turn toward the gradient is increased the advantage to the signalling phenotype declines smoothly and the cost that can be imposed on signalers before the strategy is inviable, is reduced. Since signalling enhances the ability of the group to track the resource, when individuals also have this capacity the role of social interations is less important. Individuals will still lose track of the resource, however the time between such occurrences will be reduced. When individuals are able to stay within high concentration regions for long periods, the advantage attained by bringing in signallers (that may be followed in the future) is lost.

This is just one example of a potential search mechanism, but there are other ways a lone individual could increase its uptake that would be appropriate in different environments. Indeed, it has been shown effective strategies vary considerably as the properties of the resource in question change [1]. Further to this there are many movement behaviours that may be employed in the absence of local information about the resource that may still increase patch encounter rates, even when there is no detected resource concentration or gradient (see [2] and references therein).

Although we demonstrate the effect of including only a simple gradient tracking strategy, we show the qualitative features of out model remain intact. We therefore fully expect introducing more complex individual level behaviour would lead to an equivalent result.

References

[1] Seymour, JR, Marcos, Stocker R (2009) Resource patch formation and exploitation throughout the marine microbial food web. Am Nat 173: 15-29.

[2] Bartumeus F, Da Luz M, Viswanathan G, Catalan J (2005) Animal search strategies: a quantitative random-walk analysis. Ecology 86: 3078–3087.
